# Vinardo: A Scoring Function Based on Autodock Vina Improves Scoring, Docking, and Virtual Screening

**DOI:** 10.1371/journal.pone.0155183

**Published:** 2016-05-12

**Authors:** Rodrigo Quiroga, Marcos A. Villarreal

**Affiliations:** Instituto de Investigaciones en Fisicoquímica de Córdoba (INFIQC), CONICET–Departamento de Matemática y Física, Facultad de Ciencias Químicas, Universidad Nacional de Córdoba, Ciudad Universitaria, Córdoba, Argentina; Universität Erlangen-Nürnberg, GERMANY

## Abstract

Autodock Vina is a very popular, and highly cited, open source docking program. Here we present a scoring function which we call Vinardo (Vina RaDii Optimized). Vinardo is based on Vina, and was trained through a novel approach, on state of the art datasets. We show that the traditional approach to train empirical scoring functions, using linear regression to optimize the correlation of predicted and experimental binding affinities, does not result in a function with optimal docking capabilities. On the other hand, a combination of scoring, minimization, and re-docking on carefully curated training datasets allowed us to develop a simplified scoring function with optimum docking performance. This article provides an overview of the development of the Vinardo scoring function, highlights its differences with Vina, and compares the performance of the two scoring functions in scoring, docking and virtual screening applications. Vinardo outperforms Vina in all tests performed, for all datasets analyzed. The Vinardo scoring function is available as an option within Smina, a fork of Vina, which is freely available under the GNU Public License v2.0 from http://smina.sf.net. Precompiled binaries, source code, documentation and a tutorial for using Smina to run the Vinardo scoring function are available at the same address.

## 1 Introduction

Protein-ligand docking is a computational method which attempts to predict the most probable position, orientation and conformation with which a ligand (often a small organic molecule) can bind to a protein. The binding free energy of a ligand to a protein can be predicted in different ways, and hence docking programs can be classified into one of the following three categories. 1- Force-field based 2- Empirical scoring functions 3- Knowledge-based potentials [1]. Different programs, using all three strategies, have been successfully used in many different drug discovery projects [2]. Autodock Vina [3] (referred to as Vina from here on) is the successor to Autodock 4, a highly successful docking program [4,5]. However, Vina is a different program and uses a different scoring function and global optimization algorithm. It is two orders of magnitude faster [3,6], and has shown similar or improved accuracy [3,6]. Vina is open source and is currently used by many groups worldwide for docking and virtual screening. The original paper describing Vina was published in 2010 and has now over 3000 citations according to Google Scholar.

For the estimation of ligand-receptor affinity, Vina uses an empirical scoring function which is inspired by the X-score function [7]. As stated by the authors, the nature of the scoring function is “more of a machine learning than a physics-based function” [3]. The aim of the present work was to develop a simpler scoring function based on Vina with fewer parameters and with a more physics-based character, that is, a scoring function composed of terms which are readily identified as some of the traditional terms used in force fields [2].

The Vina scoring function is implemented not only in the Vina program but also in other closely related programs as iDock and Smina [8,9]. The Smina program is a fork of Vina recently published by Koes et al. [9] which maintains most of Vina ´s functionality, and adds a myriad of new features, many of them associated with energy minimization and the possibility to easily develop new scoring functions, which makes it a very interesting tool. Smina also provides a very easy, hassle-free way of defining the search space, also called bounding box, which in Vina was not straightforward. Koes et al. [9] also developed a novel scoring function (referred to as Dk_scoring from here on), selecting energetic terms using the forward selection algorithm, and then assigning weights to each term using linear regression between experimental and predicted binding energies of the CSAR 2012 dataset [10]. The Dk_scoring function shows improved correlation between the calculated and experimental binding affinity for the training set used, as compared to Vina. On the other hand, it is apparently less efficient than Vina when used for docking, and also at predicting poses which closely resemble the crystallized protein-ligand complexes used for training [9].

We have used Smina as a tool to develop Vinardo (Vina RaDii Optimized), a scoring function which shares component terms with the Vina scoring function: steric interactions, hydrophobic interactions, and non-directional H-bonds. Despite sharing component terms, Vinardo displays several differences with Vina; a modified steric interaction term, new atomic radii, and simplified interactions (using a lower number of parameters). Vinardo is implemented as an optional scoring function in Smina. To compare the docking abilities of Vinardo and Vina, we performed re-docking assays on four high quality datasets. To measure the scoring and ranking abilities of Vinardo, we repeated the scoring function analyses performed in CASF 2013 [11]. Finally, we tested virtual screening capabilities by docking a multitude of active and inactive compounds against different proteins available in the DUD database, and verifying Vinardo's capability to rank active compounds above inactive ones.

As we will show in the following sections, Vinardo displays consistently superior docking, scoring, ranking and virtual screening capabilities for all datasets analyzed, compared to Vina and Dk_scoring.

## 2 Materials and Methods

### 2.1 Scoring Functions

#### 2.1.1 Vina

An empirical scoring function calculates the affinity, or fitness, of protein-ligand binding by summing up the contributions of a number of individual terms [1]. Each of these terms generally represent an important energetic factor in protein-ligand binding. There are several parameters involved in each of these functions which can be modified to improve the predictions. Lastly, each term is weighted (multiplied by a constant) before being summed up into the final predicted binding affinity. In the following paragraphs we give a brief description of the Vina scoring function in order to compare it to Vinardo. For a more detailed description of the Vina scoring function, the reader is referred to the original paper by Trott and Olson [3].

The binding energy is predicted as the sum of distance-dependent atom pair interactions (Eq 1).

$$E=\sum e_{pair}\;\left(d\right)$$ Eq 1

Here *d* is the surface distance calculated with Eq 2, where r is the interatomic distance and Ri and Rj are the radii of the atoms in the pair.

$$d=r-R_{i}-R_{j}$$ Eq 2

Every atom pair interacts through a steric interaction given by the first three terms of Eq 3. Also, depending on the atom type, there could be hydrophobic and non-directional H-bonding interactions, given by the last two terms of Eq 3.

$$e_{pair}\left(d\right)=\left\{ \begin{array}{c} w_{1}*{Gauss}_{1}\left(d\right)+\\ w_{2}*{Gauss}_{2}\left(d\right)+\\ w_{3}*Repulsion\left(d\right)+\\ w_{4}*Hydrophobic\left(d\right)+\\ w_{5}*HBond\left(d\right) \end{array}\right.$$ Eq 3

Steric interaction in Vina is assessed using three terms (Eqs 4 to 6). The combination of an attractive Gaussian function with a repulsive parabolic function reproduces the general shape of a typical Lennard-Jones interaction, provided the Gaussian term is negative and the parabolic positive. A graphical representation of these functions is shown in S1 Fig (also see Figure 1 in [3]). If both atoms in the pair are hydrophobic the linear function in Eq 7 is included. Also, if the pair consists of an H-bond donor and an H-bond acceptor, Eq 8 is added. These last two equations are simple piecewise linear and their effect can be thought of as modifying the steric interaction in order to produce an increased attraction when these types of interaction are present. S2 Fig shows an example of this (also see Figure 1 in [3]).

$${Gauss}_{1}=e^{-{\left(\left(d-o_{1}\right)/s_{1}\right)}^{2}}$$ Eq 4

$${Gauss}_{2}=e^{-{\left(\left(d-o_{2}\right)/s_{2}\right)}^{2}}$$ Eq 5

$$Repulsion\left(d\right)=\left\{ \begin{array}{c} d^{2}ford\leq 0\\ 0ford>0 \end{array}\right.$$ Eq 6

$$Hydrophobic\left(d\right)=\left\{ \begin{array}{c} 1ford\leq p_{1}\\ p_{2}-dforp_{1}>d<p_{2}\\ 0ford\geq p_{2} \end{array}\right.$$ Eq 7

$$HBond\left(d\right)=\left\{ \begin{array}{c} 1ford\leq h_{1}\\ \frac{d}{-h_{1}}forh_{1}<d<0\\ 0ford\geq 0 \end{array}\right.$$ Eq 8

The mechanism by which these terms were selected for the Vina scoring function, the parameters used therein, and the weight of each term are unclear, although some kind of non-linear regression on the PDBBIND 2007 database was used, since we quote the original Vina paper: “in tuning the scoring function, we went beyond linear regression” [3].

#### 2.1.2- Vinardo

We generated a variety of scoring functions which consisted of the inclusion/exclusion of several interaction terms to the Vina scoring function. The terms considered for addition or exclusion were Gaussian steric attractions, quadratic steric repulsions, Lennard-Jones potentials, electrostatic interactions, hydrophobic interactions, non-hydrophobic interactions, and non-directional hydrogen bonds. These terms are all pairwise additive and are part of the 26 currently available in the Smina program (described in detail in [9]).

The procedure followed to select the best docking function was as follows: A large set of trial scoring functions was generated by systematically exploring the vast combinatorial possibilities of individual energetic terms, parameters present in those terms, atomic radii, and weights applied to each term. The initial, final and step size values used in the exploration of the parameter space is shown in S1 Table.

1. With each trial function, we minimized the ligand in each crystallographic structure in the PDBBIND Core 2013 dataset [11] and calculated the average RMSD between the minimized ligands poses and the original crystallized structures. Due to restricted availability of computational resources, only the 20 trial functions with the lowest average RMSD were selected for further evaluation and refinement.
2. The evaluation consisted of re-docking the structures in the PDBBIND Core 2013 dataset as well as the Iridium-HT dataset.
3. The 5 trial functions with the best re-docking performance were refined by exploring small variations in weights and parameters of the function, and once again evaluating their re-docking abilities.

#### 2.1.3 –Dk_scoring

Dk_scoring is a recently developed scoring function [9]. It uses a 4–8 Lennard-Jones term to account for steric interactions. The solvation effects are treated with the same term as in Autodock 4 [5]. The function also includes hydrogen bond interactions, handled with the same equation as Vina (Eq 8). Compared to Vina and Vinardo, the steric interaction is softer. This could be a downside when performing docking, but it can be effective for energy minimizing a pose within a sub-optimal receptor (Dr. David Koes, personal communication). Dk_scoring is included in Smina as a built-in function along with Vina and Vinardo. We included Dk_scoring in this work to further test its performance beyond the analysis already carried out by Koes et al. [9].

### 2.2- Smina program

For performing docking, scoring and minimization, we used Smina [9]. Smina is a fork of AutoDock Vina [3], providing enhanced support for minimization and scoring. It is available under a GPL2 license at http://smina.sf.net. For docking, Smina was used with the “—exhaustiveness 32” option. The search space box used for docking was centered around the bound ligand pose with each dimension extended 15 Å from the ligand. Vina is the default scoring function in Smina and the “—scoring = <function_name>” option allows the use of other scoring functions such as Vinardo and Dk_scoring.

### 2.3- CASF-2013

The CASF-2013 evaluation [11] is based on the PDBBIND 2013 dataset and consists of a series of tests designed to evaluate different aspects of a scoring function. For further details of the tests the reader is referred to the original reference [11], as we have repeated the analyses performed therein. A description of the aim of the four main tests performed is provided here. 1- Scoring power, which measures the ability to predict binding affinities that linearly correlate with experimentally measured affinities. 2- Ranking power is the ability to correctly rank the binding affinity of different ligands to the same protein. 3- Docking power is the ability to select a ligand pose which closely resembles the crystallized pose as the best pose, from a set of pre-calculated, computationally generated poses. 4- Screening power is the ability to select the true best binder from a pool of varied ligands.

In running these tests, Dk_scoring, Vina and Vinardo were run in the “score-only” mode of Smina, where no docking is performed, and only the protein-ligand binding affinity of the given structure is calculated. The poses analyzed are the same poses generated and used in Li et al. [11].

### 2.4- RMSD

Symmetry-corrected RMSD was calculated using the Hungarian-algorithm-based procedure available in DOCK6 [12].

### 2.5- Preparation of structures

For all necessary file format conversion, Open Babel [13] was used. For further file preparation, like generating the pdbqt files to run Smina, we used prepare_receptor4.py and prepare_ligand4.py scripts from AutoDock Tools [4] to assign partial charges and protonation states. Vanadium atoms, which are not supported by the Autodock Tools scripts, were replaced by Sulfur atoms.

### 2.6- Molecular graphics

Molecular graphics were generated using UCSF Chimera [14], surfaces were colored according to the Kyte and Doolittle hydrophobicity scale [15].

## 3- Results

### 3.1- Development of the scoring function

**Fig 1:**
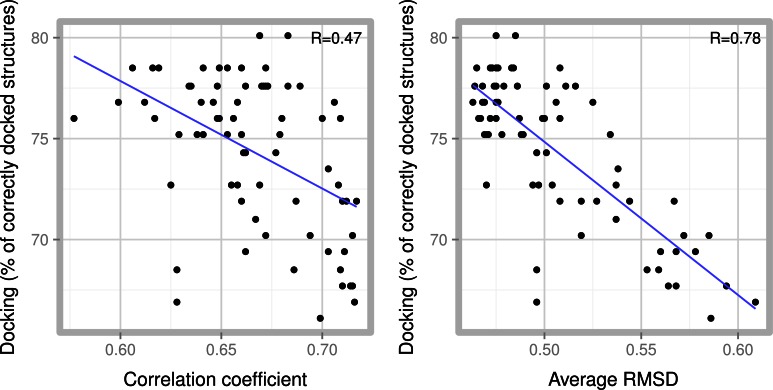
Docking ability prediction. Docking ability for 72 scoring functions generated by small perturbations of the Vina scoring function. The docking ability is measured as a percentage of the 122 complexes which the scoring function managed to dock correctly, compared to: (A) Pearson correlation coefficient between calculated and experimental binding affinities. (B) Average RMSD. (B). In blue, a linear regression of the data is shown, as a trend, and the Pearson correlation coefficient of the regression is shown as R.

A widespread observation in the field of protein-ligand prediction is that scoring functions are typically either apt at ranking crystallized ligands (scoring capability), or apt at predicting the best position and orientation of the ligand (docking capability) [11]. In this work our interest was to develop a scoring function based on the highly successful Vina scoring function, that improves its docking capabilities. To this end, a possible strategy is to select a protein-ligand dataset, define a scoring function, evaluate its capabilities in re-docking experiments and, based on this result, elaborate a new scoring function or refine its parameters. With a dataset in the order of hundreds of complexes, this methodology is not feasible due to the huge combinatorial space of possible terms and parameters involved. A common strategy (which was used in the development of both Vina and Dk_scoring) has been to train the scoring function by regression of experimental and predicted binding affinities of a selected dataset. In this way, only the calculation of the binding energy of the crystal protein-ligand structures in the dataset is performed, without the need of the more time consuming re-docking procedure. Vina was trained on the PDBBIND 2007 dataset, and Dk_scoring with the CSAR-NRC HiQ 2010 and CSAR 2012 datasets [3,9]. Regression ensures good scoring capability but not necessarily good docking capability; therefore we decided to explore the relationship between linear regression training and docking capability. The selected dataset was the PDBBIND 2013 dataset (195 structures) which is a curated protein-ligand dataset which includes experimental binding data. Due to time constraints, for this exploratory phase of the development, we reduced the size of the PDBBIND Core 2013 dataset, only retaining ligands with 7 or less rotatable bonds, which makes up a total of 122 structures. We prepared 72 scoring formulas by perturbing the Vina scoring function with small variations in the number of terms, parameters, weights, and atom radii. For each scoring formulation we calculated the correlation coefficient between experimental and calculated binding affinity (scoring ability), and compared these coefficients against re-docking ability. Re-docking ability was measured as the percentage of ligands for which the RMSD of the best scoring pose was within 2 Å of the crystallized ligand structure for each ligand present in the dataset. As shown in Fig 1A, scoring ability is a very poor predictor of the docking ability of a scoring function.

Since the output of the docking procedure in Vina is a set of energy minimized poses of the ligand, we hypothesized that minimization of the ligand in the crystal structure, instead of simply scoring, could better anticipate the docking ability of a scoring function. The rationale behind this is that having a local minimum close to the crystallographic structure is a necessary (albeit not sufficient) condition for successful docking. The condition of a local minimum close to the experimental structure is necessary since docking success is measured as the distance (in RMSD terms for example) between the calculated and the experimental structure. Nevertheless, it is not sufficient because this minimum must also be the global minimum, given that the global minimum is, ideally, the result of the docking procedure. To test this idea, taking each of the 72 functions, we minimized the ligand in each crystallographic structure in the dataset and calculated the RMSD between the minimized ligand pose and the original crystallized structure. There was significant linear correlation between the average RMSD after minimization and the docking ability of scoring functions (Fig 1B). This implies that having a local minimum close to the crystallographic structure helps to improve the docking ability. Despite the clear correlation between low average RMSD and good docking ability, they do not have a one to one relationship, and therefore there are several scoring functions with very similar RMSD but different docking accuracy.

Having established that low average RMSD values after minimization correlate with good docking ability, we performed a systematic parameter search on the full PDBBIND 2013 dataset, as described in Methods. Succinctly, this consisted of searching for combinations of interaction terms, weights and parameters, which resulted in functions with low average RMSD values. In the final step of the development, an actual docking evaluation is performed on a pool of these functions, and we selected the scoring function with the best docking performance.

The final combination of interaction terms present in Vinardo is the same as that of Vina, except for the removal of the second attractive Gaussian term (Eq 5). Vinardo also uses different atomic radii, parameters within interaction terms, and term weights. A comparison of the term weights used in Eq 3 for Vina and Vinardo are shown in Table 1. The parameters used in Eqs 4–8 are shown in Table 2. A comparison of the atom radii used in Vina and Vinardo is shown in Table 3.

**Table 1:** Weights applied to each term in Vina and Vinardo. Comparison of the weights applied to each energetic term (see Eq 3).

	*w*_*1*_	*w*_*2*_	*w*_*3*_	*w*_*4*_	*w*_*5*_
**Vina**	-0.035579	-0.005156	0.840245	-0.035069	-0.587439
**Vinardo**	-0.045	0.000	0.800	-0.035	-0.600

**Table 2:** Values for parameters used in energetic terms. Comparison of the parameters used in Eqs 4 to 8, values in Å.

	*o*_*1*_	*s*_*1*_	*o*_*2*_	*s*_*2*_	*p*_*1*_	*p*_*2*_	*h*_*1*_
**Vina**	0.0	0.5	3.0	2.0	0.5	1.5	-0.7
**Vinardo**	0.0	0.8	-	-	0.0	2.5	-0.6

**Table 3:** Atomic radii used in Vina and Vinardo scoring functions. C_A_ are aromatic carbons. Values are in Å.

	C	C_A_	N	O
**Vina**	1.9	1.9	1.8	1.7
**Vinardo**	2.0	1.9	1.7	1.6

As mentioned before, and shown in S1 Fig, the sum of a Gaussian and a quadratic function produces an energy profile which resembles a Lennard-Jones equation. The main difference between Vinardo and Vina is that in Vinardo we removed the second Gaussian which produces a second minimum in the steric interactions (see S1 Fig). This minimum is centered well outside the contact between atoms and we refer to this as a “long-range” attraction. The inclusion of this second minimum is difficult to justify from a physical point of view, although empirically it has been shown to be effective as Vina is one of the most successful docking programs available. Another difference to note between Vinardo and Vina is the change in atomic radii. This is a very sensitive parameter which influences not only steric, but every interaction, through the calculation of surface distance (Eq 2). Lastly, we note the elimination of the hydrophobic interaction onset parameter (p_1_ in Eq 7), which was fixed at zero.

### 3.2- Characterization of the Vinardo scoring function

The Vinardo and Vina scoring functions are described by Eqs 1 to 8 and their parameters shown in Tables 1–3. Although Vinardo is similar to Vina in the component terms that make up the scoring function (steric interactions, hydrophobic attraction, non-directional hydrogen bond), the combination of the simplification of the terms, differences in weights, atomic radii and parameters determine that Vinardo and Vina behave quite differently. In order to produce a more in-depth comparison of both functions, we performed an energy minimization of every ligand in the crystallographic structure in the PDBBIND Core 2013 database, and measured the average contribution to the final binding energy of each individual term. These results are shown in Table 4.

**Table 4:** Average contribution of each interaction to the final predicted binding energy. Determined for the 195 protein-ligand complexes found in the PDBBIND Core 2013 database. Values are expressed as percentages of final average binding energy.

	Steric interaction			Non Steric Interaction		
	**Lennard Jones 4–8**	Gauss1 + Repulsion	Gauss2 (long range attraction)	Hydrophobic attraction	Non- directional Hydrogen bond	Solvation
**Vina**	-	5%	58%	11%	26%	-
**Vinardo**	-	46%	-	26%	28%	-
**Dk_scoring**	103%	-	-	-	23%	-26%

As shown in this table, although the second Gaussian term in Vina is multiplied by a smaller weight compared to the first Gauss term (see Table 1), it actually contributes ten times more to the final predicted binding affinity (58%). This can be explained by the fact that a larger number of atom pairs are included in the second Gaussian due to its “long-range” nature. On the other hand, in Vinardo the steric interaction component has no “long-range” attraction term, and steric interactions as a whole represent a smaller percentage of the predicted binding energy. Additionally, the contribution to the binding energy of the hydrophobic term in Vinardo is more than double that of Vina, due to the increased offset of the interaction (parameter p2 in Eq 7). Table 4 also shows the analysis of the Dk_scoring function. In this case, the binding energy is largely dominated by the steric interaction term, handled with a 4–8 Lennard-Jones potential. It is also interesting to note that the Autodock 4 solvation term, on average, opposes the binding of the ligands. This could be indicative of a poor account of the hydrophobic interactions.

### 3.3- Vinardo displays improved docking

We tested Dk_scoring, Vina and Vinardo for their ability to correctly re-dock the ligands present in four different datasets. The first dataset analyzed is Iridium-HT [16], which was also used in the final steps of Vinardo development (see Materials and Methods, section 4.1.2). The other three chosen datasets were CSAR 2012, Iridium-MT (the complexes deemed as “moderately trustworthy” by Warren et al. [16]), and Astex-diverse [17]. We generated 10 different solutions for each ligand, and analyzed whether the top 1, top 3, or top 5 poses (sorted by predicted binding affinity) were successfully docked. A docking run was considered to be successful when the RMSD between the predicted pose and the crystallographic position of the ligand was equal to, or less than, 2 Å.

**Fig 2:**
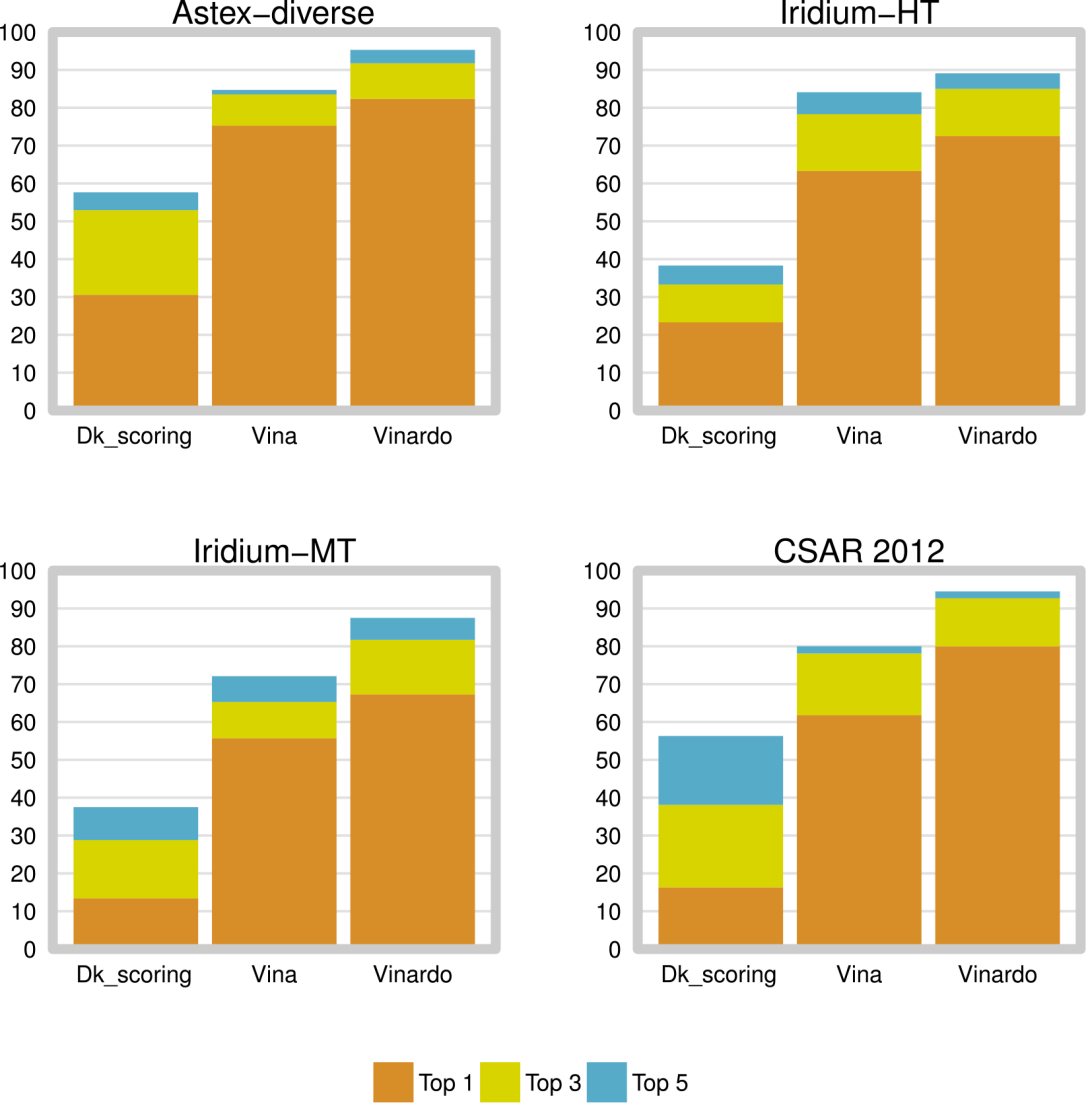
Docking performance for 4 different datasets. Docking performance of Dk_scoring, Vina and Vinardo was assessed for all protein-ligand complexes in the Astex-diverse, Iridium-HT, Iridium-MT, and CSAR 2012 datasets. Results are displayed as percentage of total compounds in the dataset for which at least one pose was correctly docked (RMSD equal or lower than 2 Å with respect to the crystallized ligand pose) and ranked by the scoring function as a Top 1, Top 3 or Top 5 pose.

As seen in Fig 2, Vinardo displays improved docking capabilities as compared to Vina and Dk_scoring. It is interesting to mention that the biggest improvement is observed in the datasets which were not used in Vinardo development, Astex-diverse, CSAR 2012 (which is the Dk_scoring training set) and Iridium-MT, which suggests an absence of over-training for Vinardo.

For ligands correctly docked by both Vina and Vinardo (189 structures in the four datasets analyzed), we compared the binding energy of the top scoring pose, with the highest affinity false positive pose. A pose was considered as false positive when the RMSD compared to the top pose was above 2 Å. The average difference increased from 0.95 kcal for Vina to 1.28 kcal for Vinardo, indicating an improved discrimination between true and false positives for Vinardo.

**Fig 3:**
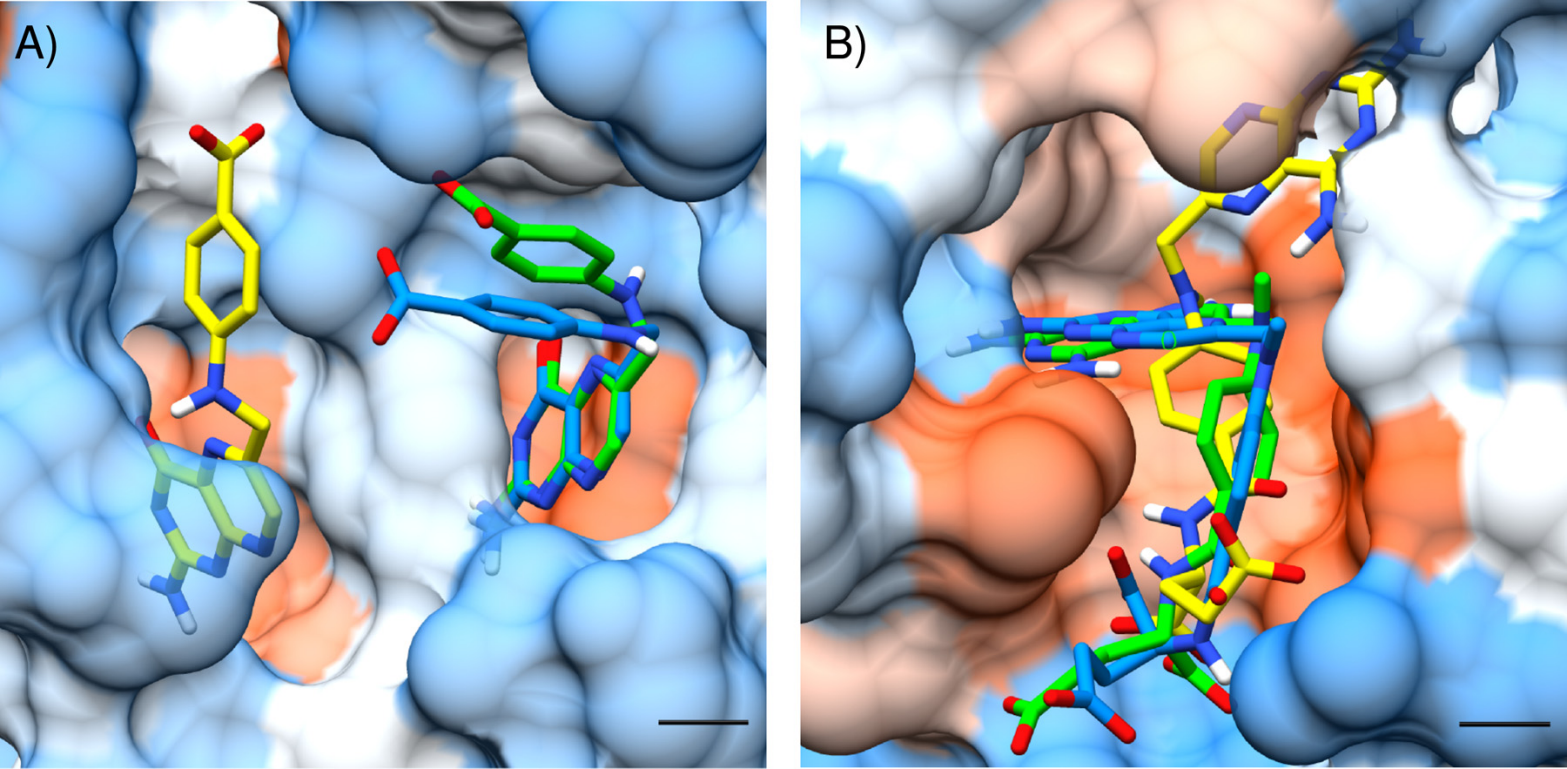
The long range steric term in Vina causes some ligands to be incorrectly docked. Ligands are depicted as sticks, with hydrogens colored in white, oxygen in red, nitrogen in blue, and carbons colored according to the origin of the ligand pose. The crystallized ligand pose is shown in cyan, the Vinardo top ranked pose in green, and the Vina top ranked pose in yellow. Scale bar corresponds to a distance of 2 Å. Protein surface is colored according to amino acid hydrophobicity, using the Kyte-Doolittle scale with colors ranging from dodger blue for the most hydrophilic, white for neutral residues, and orange red for the most hydrophobic residues.

As mentioned before, Vina´s main energetic term is the long-range steric attraction term. This tends to produce binding poses deeply buried in the binding pocket, in order to maximize the number of protein-ligand contacts. We have noticed that this is the cause for Vina incorrectly predicting the most stable binding pose of many structures. As an example, in Fig 3, we show the crystallized ligand structure (cyan), as well as the highest affinity pose predicted with Vina (yellow) and Vinardo (green) for 1br6 (Fig 3A) and 1dss (Fig 3B) (both from Iridium-HT dataset). In both cases Vina incorrectly places ligand atoms in the deepest pocket available, which leads to incorrect results.

### 3.4- Vinardo as a scoring function—CASF 2013 evaluation

In addition to testing the ability of Vinardo to dock ligands successfully, we evaluated how well it performs as a scoring function. To this end, we decided to repeat the tests performed in the CASF-2013 evaluation by Li et al. [11], which would allow the comparison of Dk_scoring, Vina and Vinardo to the myriad of open source and commercial scoring functions tested therein, providing a frame of reference for our comparison of Vina and Vinardo. We first started by repeating the tests by Li et al. [11] with X-Score, in order to validate our implementation of the different metrics used in the mentioned work. Our results are in excellent agreement with the published results, making us confident of any direct comparison between the scoring functions we tested and those reported by Li et al. [11]. Our comparison of Dk_scoring, Vina and Vinardo is restricted to only the three top scoring functions from CASF-2013. These are X-Score [7], Glidescore-SP [18] from the Schrodinger software package, and ChemPLP [19] from the GOLD software package [20]. It is interesting to note that Vina is inspired by the X-Score function [3].

#### 3.4.1- Scoring power

Scoring power measures the ability to predict binding affinities that linearly correlate with experimentally measured affinities. The Pearson correlation coefficient between calculated and experimental binding affinities for each scoring function is reported in Table 5. Additionally, as it is not included in the original CASF 2013 test, for Dk_score, Vina and Vinardo, we calculated correlation using energy-minimized structures, as well as the average RMSD of the minimized ligand poses with respect to the crystal structure. Vinardo is second only to X-score in Scoring power, and it achieves a correlation of 0.601 for the CASF-2013 dataset. After energy minimization, the correlation of all the tested scoring functions increased. This would indicate that the predicted binding energy of the energy minimized structure is a better measure of the real Scoring power of a function. Interestingly, Dk_score displays an improved correlation with respect to Vina. However, when minimization is performed using Dk_score, the resulting structures are more divergent as compared to the crystallized ligand, than when Vina or Vinardo are used in the minimization. This result is in line with the observation made before (see Fig 1), that scoring functions with high RMSD values after minimization tend to perform worse at docking, as Dk_scoring displays less docking aptitude than Vina and Vinardo (see Fig 2).

**Table 5:** Scoring power for each scoring function, as defined in CASF 2013. Correlation coefficients between experimental and calculated binding affinity. In column labeled “R”, correlation coefficients are calculated by scoring crystal structures. In column labeled “Minimized-R”, energy minimization is performed before the calculation of correlation coefficients. The average RMSD of minimized structures with respect to crystallized ligands are shown in column “RMSD” (values are in Å). Results for GlideScore-SP, ChemPLP@GOLD and X-Score are taken from [11].

Scoring Function	R	Minimized-R	RMSD
**X-score**	0.614		
**Vinardo**	0.601	0.625	0.514
**ChemPLP@GOLD**	0.594		
**Dk_scoring**	0.583	0.614	0.896
**Vina**	0.564	0.607	0.576
**Glidescore-SP**	0.452		

**Fig 4:**
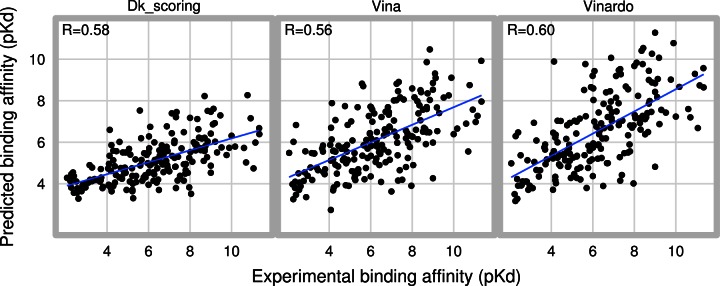
Scatter plots comparing predicted binding affinities to experimental binding affinities. Predicted binding affinities were calculated using each scoring function on the crystallized poses for the PDBBIND Core 2013 database. In red, the result of a linear regression is shown.

Fig 4 shows a scatter plot comparing predicted and experimental binding affinities for all 195 protein-ligand complexes in the PDBBIND Core 2013 database. Vinardo displays the highest correlation, and also the highest slope, although the slope is still far less than the ideal slope value, of 1.

#### 3.4.2- Ranking power

Ranking power measures the ability to correctly rank the binding affinity of different ligands to the same protein. Three ligands which bind to each protein are ranked by the scoring functions. “High success” is defined when all three ligands are correctly ranked according to their experimental binding energies. “Low success” is defined when only the top ligand is correctly ranked. Table 6 shows that Vinardo is the most successful among all the tested scoring functions, correctly ranking all three ligands for 45 (70.8%) of the 65 proteins present in PDBBIND Core 2013, as compared to 38 proteins (58.5%) for the most successful scoring functions in CASF-2013 for this test which was a tie between X-Score and ChemPLP@GOLD. As far as measuring “Low success”, Vinardo correctly ranks 45 out of 65 top binding ligands (70.8%), while Vina, Dk_scoring, X-score and ChemPLP@GOLD correctly rank 46 out of 65 top binding ligands (72.3%).

**Table 6:** Ranking power for each scoring function, as defined in CASF 2013. The 195 structures in PDBBIND Core 2013 database consist of 65 proteins with three ligands each. Ranking power is measured as “High success” when all three ligands are correctly ranked, or “Low success”, when only the top ligand is correctly ranked. Values are reported as percentages of the total 65 proteins. Results for GlideScore-SP, ChemPLP@GOLD and X-Score are taken from [11].

Scoring Function	High	Low
**Vinardo**	70.8	70.8
**Vina**	63.1	72.3
**Dk_scoring**	58.5	72.3
**X-Score**	58.5	72.3
**ChemPLP@GOLD**	58.5	72.3
**GlideScore-SP**	43.1	56.9

#### 3.4.3- Docking power

Docking power is the ability to select a ligand pose which closely resembles the crystallized pose as the best pose, from a set of pre-calculated, computationally generated poses. This is evaluated by scoring 50 to 100 different poses for each ligand (refer to the CASF-2013 paper [11] for details on how these poses were selected), and verifying if the scoring function ranks a pose with RMSD within 2 Å of the crystallized pose, as top1, top2, or top3 poses in predicted affinity. Vinardo once again is the most successful function by a large margin. It should be mentioned that CASF-2013 used an unpublished method to calculate “symmetry-corrected RMSD” values, while we used the Hungarian-algorithm-based symmetry corrected RMSD used in DOCK6 [12], so small differences in the results could be due to this fact. Nevertheless, our own tests using X-score show quasi-identical results to Li et al. As shown in Table 7, Vinardo ranks correctly docked poses as the top scoring pose for 175 (89.7%) of the 195 ligands in PDBBIND Core 2013. It is followed by Vina, which performs similarly to ChemPLP@GOLD, which was the most successful scoring function for this test in CASF-2013.

**Table 7:** Docking power for each scoring function, as defined in CASF 2013. Each scoring function was used to measure the energy of many different poses for each protein-ligand complex. A scoring function was considered successful when the top scoring pose was equal to or lower than 2 Å from the crystallized pose. Values are reported as percentages of the total 195 protein-ligand complexes analyzed. Results for GlideScore-SP, ChemPLP@GOLD and X-Score are taken from [11].

Scoring Function	top1	top2	top3
**Vinardo**	89.7	92.8	94.4
**Vina**	80.5	88.2	90.8
**ChemPLP@GOLD**	82.1	87.2	90.3
**GlideScore-SP**	78.9	86.2	88.7
**Dk_scoring**	66.7	78.9	82.1
**X-Score**	63.0	75.0	79.0

#### 3.4.4- Screening Power

Screening power determines whether the scoring functions are capable of identifying the true binder for the 65 proteins in the dataset, from a pool of 195 ligands. For each protein in the dataset, 50–100 poses of each compound were analyzed to determine the most negative energy pose for each compound, to each protein. Then, the 195 compounds were ranked according to the score assigned by the scoring function. If a scoring function placed the known best binder of a protein in the top 1%, top 3% and/or top 5% of compounds, that scoring function was considered successful for said protein. In a way, this test determines how well the scoring function is able to compare the predicted binding affinities of different ligands to the same protein.

Once again, the most successful scoring function is Vinardo, which manages an impressive level of success, as observed in Table 8. Vinardo is able to identify the true best binder as the first or second best ligand (top 1%) amongst the pool of 195 ligands for 52 of the 65 proteins (80%). The best performing scoring function for the Screening Power test in CASF 2013 was GlideScore-SP with a success rate of 60% for the top 1% of ligands. Vina correctly selects the true binder for 53.8% of proteins, which puts it right behind GlideScore-SP in the Screening Power test, but far behind Vinardo. This suggests Vinardo is more capable of correctly predicting the binding affinities of false and true binders, which makes it more suitable than Vina for performing virtual screenings.

**Table 8:** Screening power for each scoring function, as defined in CASF 2013. Values are reported as percentages of the total 65 proteins for which each scoring function was successful (the known true binder of said protein was ranked in the top 1%, top 3% or top 5% of the 195 compounds tested). Results for GlideScore-SP, ChemPLP@GOLD and X-Score are taken from [11].

Scoring Function	top1%	top3%	top5%
**Vinardo**	80.0%	89.2%	89.2%
**GlideScore-SP**	60.0%	72.3%	76.9%
**Vina**	53.8%	70.8%	78.5%
**ChemPLP@GOLD**	41.5%	70.8%	84.6%
**Dk_scoring**	16.9%	38.5%	46.2%
**X-Score**	9.23%	21.5%	32.3%

### 3.5- Virtual Screening—DUD dataset

As a further measure of the virtual screening capabilities of Dk_scoring, Vina, and Vinardo, we performed a virtual screening of the DUD library [21] using docking. The HIV protease in the DUD library has been used previously to compare the virtual screening capabilities of Vina and Autodock 4, where Vina was found to be more effective than Autodock 4 [6]. The DUD library is very extensive, with a total of 2950 active compounds and nearly 100000 decoys compounds for 40 protein targets. Docking all these compounds to their targets would require massive amounts of CPU time, so we decided to analyze all targets where the number of total compounds was 3000 or less. This choice leaves a total of more than 40000 compounds for 28 protein targets analyzed. The chosen targets for virtual screening by docking are shown in S2 Table.

**Fig 5:**
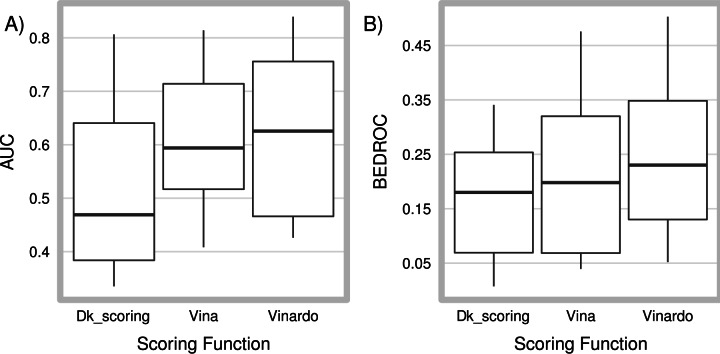
Overall and early enrichment of active compounds from the DUD dataset. 28 proteins from the DUD dataset were used to dock ligands and decoys. A thick black line represents the median value, the box limits represent the first and third quartile, while whiskers represent the 9^th^ and 91^st^ percentile. (A) AUC is used as a measure of overall enrichment. (B) BEDROC is used as a measure of early enrichment. For protein comt, the cofactor molecule SAM was removed from the original DUD pdb file, since many inhibitors bind to the cofactor binding site.

For each target, we docked all compounds using the three different scoring functions, and determined the area under the receiver operating characteristic (ROC) curve (AUC) as a way of measuring overall enrichment of active compounds, as well as the BEDROC metric, as a measure of early enrichment of active compounds. AUC values range from 0 to 1, with 0.5 being the expected value for random selection of compounds. BEDROC values vary from 0 to 1 by definition, and a value of 0.05 is expected for random selection of data. The detailed results are shown in S3 Table and S4 Table. In Fig 5 these results are shown as a boxplot, where the median is shown as a thick black line, boxes represent the first and third quartiles, and whiskers represent the ninth and ninety-first percentiles. In this test, Vinardo showed improved performance as compared to Vina and Dk_scoring in both overall and early enrichment.

As expected, since docking is not always the optimum strategy for virtual screening [22], for some proteins none of the scoring functions performed well (Entries “comt”, “ampc”, “ace”, and “na” in S2–S4 Tables). However, for other targets (Entries “gart”, “hivpr”, “vegfr2” and “alr2” in S2–S4 Tables) Vinardo managed to outperform not only Vina and Dk_scoring, but also all the ligand-based virtual screening programs analyzed in Venkatraman et al. [23]. Lastly, although Dk_scoring performs worse than random selection of compounds at overall enrichment (median AUC of 0.469), it displays good early enrichment, and most interestingly, it performs very well on some targets where all other methods analyzed here or in Venkatraman et al. [23] perform badly, such as “pr” and “trypsin” targets (see S4 Table). This suggests that designing scoring functions specifically for a given protein target could result in optimal virtual screenings [24].

## 4- Discussion

A typical training of empirical scoring functions is performed through some kind of regression between experimental and predicted binding affinities of a dataset of protein-ligand complexes [1,2]. We found that this could lead to functions with good scoring capabilities but sub-optimal docking capabilities. A better, and equally simple strategy to easily anticipate the docking capabilities of a scoring function, is to perform an energy minimization of the crystallized ligand in the context of the receptor, followed by computing the RMSD, and averaging the results over the entire dataset. As shown in Fig 1, the lower this average RMSD is, the better the docking ability of the scoring function. Since minimization consumes only a fraction of the computer time that actual docking does, with this strategy it is possible to explore a large parameter space, which is crucial in the initial steps of developing a new scoring function. After this initial exploration it is possible to perform actual re-docking runs in interesting zones of the parameter space selected by minimization. Re-docking runs are needed because low RMSD values after minimization ensure a local minimum close to the crystalline structure, but only docking can assess if this local minimum also corresponds to a global minimum.

With this optimization strategy we were able to arrive at a scoring function which is simpler than Vina, and at the same time improves the docking, scoring, ranking and virtual screening results of this already successful scoring function. The main difference in Vinardo is the elimination of the physically unsound second minimum in the steric interaction of Vina. This second minimum, centered at 3 Å away from the sum of the atom pair radii, has the effect of producing poses which are highly buried in the binding pocket (Fig 3). An analysis of the contribution of the different terms to the binding energy in the PDBBIND 2013 dataset showed that in Vina this second minimum is the main contributing term to the final binding energy. For Vinardo, the steric term has a single minimum centered at the sum of the atom pair radii.

In developing our scoring function we noted that the atomic radii were one of the most important variables that improved docking performance. Compared to Vina, Vinardo uses increased atomic radius for aliphatic Carbons and decreased radius for both Nitrogens and Oxygens (Table 3). Overall, both scoring functions use a small set of atomic radii. For example a generic force field for organic molecules like DRIEDING [25] defines a total of 11 different atomic radii for C, N and O, while Vina uses only three and Vinardo four. This would indicate that using a more specialized set of atomic radii could improve the performance of scoring functions even further.

The steric interactions in Vinardo are treated differently than in typical force-fields. Vina and Vinardo use a combination of a Gaussian attraction and a quadratic repulsion, while typical force fields use a Lennard-Jones potential. For example, Dk_scoring and X-score use a 4–8 Lennard-Jones type potential, while DOCK [26] employs a more traditional 6–12 form. During early stages of Vinardo development, we attempted to use Lennard-Jones type potentials for steric interactions going as far as testing many different exponents, but we were not able to find a Lennard-Jones type of formulation that outperformed the Gaussian attraction plus quadratic repulsion.

Another change worth noting is that the hydrophobic term in Vinardo has only one parameter (p2 = 2.5 Å in Table 3), which roughly corresponds to the diameter of a water molecule. In this way a hydrophobic interaction is accounted for from the contact of the atomic radii, decreasing linearly until a distance where a water molecule can be placed in between the interacting pair.

In the exploration of the parameter space we found a very degenerated landscape. For example it is possible to find different sets of weights w_i_ in Eq 3 that produce practically the same final results. This means that there is no need to perform a fine grid search and go beyond two or three decimal places in defining the different parameters. This relieves some of the computational burden of searching the parameter space.

Through all the tests performed, which included scoring, ranking, and docking, for all datasets analyzed Vinardo consistently showed improved performance with respect to Vina and Dk_scoring. Vinardo also outperformed the best scoring functions tested by Li et al. in CASF 2013 [11]. It must be noted that the CASF 2013 evaluation is based on the PDBBIND Core 2013 dataset which is one of the two datasets used to develop and train Vinardo. Nevertheless, it must also be noted that none of the tests in CASF 2013 was used in the development or training of Vinardo. We developed the function by optimizing docking capability, while the tests in CASF 2013 are more oriented to re-scoring. This is an important difference, since CASF 2013 tests consist of scoring the predefined poses that conform the dataset, while in docking runs, a self-consistent exploration of the configuration space is performed.

Vina has been used by many groups to perform virtual screenings [24,27,28]. Based on the results obtained for the DUD database, we recommend the use of Vinardo instead of Vina for performing virtual screenings. Vinardo was superior to Vina both in early and overall enrichment of active compounds (see Fig 5, Table 8, S3 Table and S4 Table in Supporting Information). Although docking is not always the optimal method to perform virtual screenings, and it shows poor results for some proteins, docking can identify scaffolds which would be completely missed by using other methods [22,29]. Recently Li et al. [8] have developed iDock, which is a virtual screening tool based on Vina, with significant speed up and similar accuracy. This lead to the later development of iStar [30], a web platform for Large-Scale Protein-Ligand Docking based on iDock. We consider that iDock and iStar would probably benefit from using the Vinardo scoring function in place of the Vina scoring function, although the posterior re-scoring of results using RF-Score performed in iDock might mitigate differences between using Vina or Vinardo.

## 5- Conclusions

This work has resulted in a simplified scoring function named Vinardo, which is based on Vina. Vinardo showed improved scoring, ranking, docking and virtual screening capabilities compared to Vina. Vinardo is available within the Smina package, as an optional built-in scoring function.

## Supporting Information

S1 Fig (A) Steric interaction between two aromatic carbons in Vinardo (black). The red curve is the Gaussian attraction, while the green curve is for the quadratic repulsion. (B) Comparison of the steric interaction between two aromatic carbons in Vina (black) and Vinardo (red). Note the presence of the second minimum at 6.8 Å in the Vina interaction.
(EPS)

S2 Fig (A) Comparison between the interaction between two Carbons without (black) and with (red) the inclusion of the hydrophobic interaction in Vinardo. (B) Comparison of the interaction between an Oxygen and a Nitrogen atom without (black) and with (red) the inclusion of the hydrogen bond term in Vinardo.
(EPS)

S1 Table Initial, final and step size values for each parameter and weight used in the systematic search. Refer to Eqs 3 to 8 in the main text for a description of each parameter and weight. The following additional conditions were applied for atomic radii: C ≥ C_A,_ N > O, and (N-O) ≤ 0.10 Å.
(XLS)

S2 Table List of DUD proteins analyzed. Target name, accession number, number of active ligands, and number of decoys for all DUD proteins analyzed in this work.
(XLS)

S3 Table Overall enrichment of active compounds (AUC values). Area Under ROC Curve (AUC) values were determined for each protein-ligand-decoy set tested, from the DUD database. Values smaller than 0.5 (which is the AUC value expected for a random selection of compounds) are in italics. Values above 0.70 are in bold.
(XLS)

S4 Table Early enrichment of active compounds (BEDROC). Early enrichment was measured for each scoring function using Boltzmann Enhanced Discrimination of ROC curve (BEDROC) values, for each protein-ligand-decoy set tested from the DUD database. Values smaller than 0.05 (value expected for random selection of compounds) are in italics. Values above 0.30 are in bold.
(XLS)
